# Utilization rate and usage patterns of phakic and pseudophakic donor corneas recovered by the Singapore Eye Bank

**DOI:** 10.1371/journal.pone.0260523

**Published:** 2021-12-02

**Authors:** Sai Kyauk, Howard Y. Cajucom-Uy, Hla Myint Htoon, Z. Zaw Htoi Aung, Jodhbir S. Mehta, Arundhati Anshu

**Affiliations:** 1 Singapore Eye Bank, Singapore, Singapore; 2 Singapore National Eye Centre, Singapore, Singapore; 3 Singapore Eye Research Institute, Singapore, Singapore; Singapore Eye Research Institute, SINGAPORE

## Abstract

**Purpose:**

To compare the utilization rate and usage patterns of pseudophakic and phakic donor corneas recovered by the Singapore Eye Bank.

**Methods:**

Records of local donor corneas recovered by the Singapore Eye Bank from 2012 to 2017 were examined. Corneas that were deemed suitable for clinical use were stratified into phakic and pseudophakic groups. We examined the basic demographic pattern of both groups and the initial type of surgery/ies that the corneas were suitable for based on tissue parameters such as time from harvesting, stromal clarity, the clear central corneal area, the presence of Descemet’s membrane tears or defects, and endothelial cell density and quality. We also identified the types of corneal grafts that the corneas were eventually used for; Penetrating Keratoplasty (PK), Anterior Lamellar Keratoplasty (ALK), Endothelial Keratoplasty (EK). Finally, the overall utilization rates for each group were determined.

**Results:**

A total of 986 corneas deemed suitable for transplant were analyzed, 908 (92%) were phakic and 78 were pseudophakic (8%). The average age of pseudophakic donor corneas was (65 ± 8 yrs. old) and there was a slight male preponderance for both groups (55%). Age adjusted analysis of pseudophakic corneas showed the endothelial cell density (ECD) (mean: 2327 ± 47.1 cells/mm^2^) and clear area (mean: 7.0 ± 0.7 mm) were lesser than phakic corneas. The percentage of pseudophakic corneas that were of EK standard (ECD >2500 cells/mm^2^) were lower compared to phakic corneas (37% and 77% respectively, p < 0.001). There was significant correlation between previous cataract surgery and the endothelial cell count of the donor corneas (p < 0.001), and regression analysis also showed a strong association of ECD with cataract surgery in reference to non-cataract surgery (-478.8 (95% CI-576.9 to -380.7). The overall utilization rate for pseudophakic corneas was 58% compared to that of phakic corneas at 83%. The most common reason for pseudophakic corneas not to be utilized was due to the presence of Descemet’s membrane (DM) tears or defects under the main or side port incision created during phacoemulsification (30%). Phakic corneas were used primarily for optical grafts 84% (mainly EK) while pseudophakic corneas were used mostly for therapeutic/tectonic grafts 47% (mainly ALK or patch grafts).

**Conclusion:**

Compared to phakic donor corneas, pseudophakic corneas generally have lower overall tissue quality leading to lower uptake by surgeons and lower utilization rates. Eye banks must continuously refine their donor acceptance criteria and engage surgeons to optimize utilization of each recovered tissue.

## Introduction

Over 2640 corneal transplants were performed in Singapore from 2012 to 2017 of which 986 corneas (37%) were from local donors and the remainder from overseas eye banks. Reflecting the progressive attitudes and advanced level of corneal transplant capabilities in Singapore, 82% were selective lamellar corneal transplantations. With an average annual throughput of 173 corneas, the Singapore Eye Bank is classified as a small-scale eye bank based on the 2016 Eye Bank Association of America Statistical Benchmarking Report [1]. Small scale eye banks lack economies of scale and are therefore acutely sensitive to overhead and recurring costs that impact their financial sustainability. It is vitally important for these eye banks to ensure that the transplant potential of each recovered cornea is maximized. By analyzing past records of potentially transplantable but unmatched corneas, eye banks are better able to understand and modify their acceptance criteria to address this problem.

Previous cataract surgery in itself is not a contraindication to cornea donation, Best *et al* stated that for penetrating keratoplasty (PK), pseudophakic donors were suitable if they met all quality criteria [2]. However, there have also been studies that have shown that cataract surgery, specifically phacoemulsification, induces endothelial cell damage by lens fragment collision, free radical formation, IOP changes, and increased temperature in the aqueous humor [3]. Several studies have consistently demonstrated lower endothelial cell densities for pseudophakic eyes compared to phakic eyes [4, 5]. Kwon *et al* and Singh *et al* stated that cataract surgery is a risk factor for low donor endothelial cell count, which may in turn affect the overall transplant potential of the donor cornea [6, 7]. Our own experience has shown that many surgeons are hesitant to use pseudophakic corneas for their elective optical grafts, and this hinders the potential utilization of corneas donated in good faith.

The proportion of blindness due to cataract in South East Asia is 42% and the calculated prevalence rate of cataract in Singapore for the year 2020 was approximately 895,000 with a projected increase of up to 1.3 Million by 2040 [8, 9]. With a cataract surgery rate (CSR) comparable to most high-income countries, the current annual volume of 35,000 cataract surgeries in Singapore is expected to increase exponentially in tandem with the ageing population, owing in large part to one of the highest life expectancies in the world [10, 11]. This will have significant implications on the percentage of pseudophakic corneas within the potential donor pool. The question of whether the lens status of the donor affects graft outcome or is used as a determinant for acceptability has been an area of considerable discussion and debate [3, 12–15]. Our study aimed to compare endothelial cell density and central stromal clear zones of phakic and pseudophakic corneas and correlate these parameters with the overall transplant utilization rate. We also examined if there was any propensity for a particular group to be used for a specific type of corneal graft.

## Materials and methods

Corneas recovered by our eye bank from 2012–2017 which passed medical, serologic and quality screening and were released for clinical use were stratified into phakic and pseudophakic groups. Data captured included donor demographics, death–to- preservation (DTP) and death-to surgery intervals (DTS), clear area and endothelial cell density (ECD).

Corneas in our eye bank were assigned a specific tissue grading (Grade A or B) based on epithelial integrity, stromal clarity, central clear corneal diameter, presence of Descemet’s membrane (DM) pathology, and endothelial cell density and quality. The details of the classification are described in Table 1. The initial assigned grading of the corneas was thus included in our analysis as we wanted to determine if there was a correlation between the initial cornea grade and the eventual outcome of the tissue for both groups. The disposition of the corneas was examined by identifying what types of corneal grafts the corneas were used for PK, Anterior Lamellar Keratoplasty (ALK), Endothelial Keratoplasty (EK) as well as the overall transplant and discard rates. For the ease for analysis, all ALK including tectonic or therapeutic patch grafts are included in the ALK group.

**Table 1:** Singapore Eye Bank cornea grading system.

Grade	Type of graft	DTS (days)	ECD (cells/mm^2^)	Clear Area >8 mm	Absence of pathology on anterior stroma	Absence of pathology on Descemet’s/ endothelium
**A**	Optical PK	Up to 7	at least 2250	YES	YES	YES
	EK	Up to 7	at least 2500	NA	NA	YES
**B**	Optical ALK	> 7	<2250	YES	YES	NA
	Tectonic, Therapeutic ALK/PK/patch grafts	>7	<2250	NA	NA	NA
**A to B**	*Corneas that did not meet grade A criteria upon re-evaluation*					

### Statistical analysis

Statistical analysis included Student’s t-test, which was used to compare the means for continuous variables between the study groups and Chi-square test which were used for categorical variables. Analysis of Covariance (ANCOVA) was conducted to determine the effect of age-adjusted dependent variables on endothelial cell quality. A regression analysis included univariate and multivariate regression models was used to examine the effect of independent variables on risk factors. The estimate of odds ratio and its relative 95% confidence interval were calculated. IBM Statistical Package for the Social Sciences version 24.0 (IBM Corp.Armonk, NY) was used to analyze the data. P < 0.05 was defined as statistically significant.

## Results

### Donor demographics

While the most common age range for both study groups were in the 60–70 year band, the average age for pseudophakic patients were higher than their phakic counterparts (65 ± 8 years vs 57 ± 12 years, p < 0.001) Table 2. Gender distribution showed a slight male preponderance for both groups but the disparity was not statistically significant. The most common causes of death (COD) were similar for both groups, namely, cancer (43%) cerebrovascular disease (22%), and cardiovascular disease (16%). Adjusting for age, the average ECD (2327 ± 47 cells/mm^2^ vs 2734 ± 13 cells/mm^2^, p < 0.001) and clear area (7.0 ± 0.7 mm vs 7.6 ± 1 mm, p < 0.001) were noted to be significantly lower in pseudophakic corneas. Pseudophakic corneas took longer to be utilized for transplant (DTS = 7 ± 4 days vs 5.7 ± 3 days, p = 0.034). Corneas that had not been utilized by Day 14 post-recovery are frozen in a minus 80 Celsius scientific freezer and kept for an additional two years for tectonic or therapeutic grafts in instances where no fresh alternative is available. Even among this group, pseudophakic corneas took longer to be utilized for transplant than phakic corneas (average 81 ± 199 days compare to 36 ± 128 days respectively, p = 0.142). DTP interval (p = 0.243) as well as COD (p = 0.951) were not significant factors affecting overall cornea quality.

**Table 2:** Baseline data of phakic and pseudophakic corneas.

		Phakic Corneas	Pseudophakic Corneas	Statistical Test
**Age**	Average Age: Yr.; ± SD; (range)	57 ± 12 (9–78)	65 ± 8 (48–76)	<0.001
**Gender**	Male: Female n (%)	500 (55%):408 (45%)	43 (55%):35 (45%)	0.992
**DTP**	Average DTP: hr: mins	3 hr 02 mins	3 hr 38 mins	0.003
**DTS**	Average DTS: days; ± SD	36 days ± 128	81 days ± 199	0.142
	*(including frozen)*			
	Average DTS: days; ± SD	5.7 days ± 3	7 days ± 4	0.034
	*(not including frozen)*			
**ECD**	ECD >2250 cells/mm^2^: n (%)	801 (89%)	41 (53%)	<0.001
	ECD >2500 cells/mm^2^: n (%)	701 (77%)	29 (37%)	<0.001
	Age adjusted average ECD: cell/mm^2^ ± SD	2734 ± 13	2327 ± 47	<0.001
**Clear Area**	Clear Area >8mm: n (%)	421 (46%)	13 (17%)	<0.001
	Age adjusted average Clear Area: mm ± SD	7.6 ± 1	7.0 ± 0.7	<0.001
**Grading**	A	615 (68%)	16 (20%)	<0.001
	A to B	90 (10%)	14 (18%)	0.027
	B	203 (22%)	48 (62%)	<0.001
**Utilization Status**	Suitable for Transplant: n	908	78	<0.001
	Frozen Corneas: n (%)	182 (20%)	41 (53%)	<0.001
	Total Transplanted: n (%)	754 (83%)	45 (58%)	<0.001
	*(including frozen corneas)*			
**Type of Corneal Graft**	EK: n (%)	305 (41%)	9 (20%)	0.006
	ALK: n (%)	259 (34%)	29 (64%)	<0.001
	PK: n (%)	190 (25%)	7 (16%)	0.146
**Type of Surgery by Indication**	Optical: n (%)	636 (84%)	24 (53%)	<0.001
	Therapeutic/Tectonic: n (%)	118 (16%)	21 (47%)	<0.001

DTP = Death to Preservation Interval, DTS = Death to Surgery Interval, ECD = endothelial cell density, Clear Area = Clear zone of cornea which is devoid of any opacity, pathology and surgical incision, A = Excellent to good stromal quality with endothelial cell density greater than 2250 cells/mm^2^. A to B = Cornea downgraded due to significant deterioration in endothelial cell quality. B = Fair stromal quality with endothelial cell density less than 2250 cells/mm^2^ and death from recovery beyond 7 days, Frozen Corneas = Storage at -80’C for long term preservation, EK = Endothelial Keratoplasty, ALK = Anterior Lamellar Keratoplasty, PK = Penetrating Keratoplasty

### Donor tissue quality (endothelial cell density and clear area)

Our eye bank policy stipulates a minimum endothelial cell density of 2250 cells/mm^2^ for a cornea to be eligible for elective optical penetrating keratoplasty and at least 2500 cell/mm^2^ for endothelial keratoplasty. 89% of phakic corneas in our series had ECD > 2250 cells/mm^2^ compared to 53% for pseudophakic corneas (p < 0.001) and similarly, 77% of phakic corneas had ECD > 2500 cells /mm^2^ compared to 37% in the pseudophakic group (p < 0.001) Table 3. Applying univariate and multivariate regression analysis of risk factors affecting endothelial cell degeneration, we were able to demonstrate that increased donor age (p < 0.001), longer death- to- surgery intervals (p < 0.001) and previous cataract surgery (p < 0.001) were statistically significant determinants Table 4.

**Table 3:** Association between tissue utilization and variable factors.

	Phakic Utilized	Pseudophakic Utilized	Statistical Test
**ECD >2500cells/mm** ^**2**^	615 (88%)	18 (62%)	<0.001
**ECD >2250cells/mm**^**2**^ **or CA >8mm**	369 (80%)	21 (55%)	<0.001
**ECD >2250 and Clear Area >8mm**	344 (90%)	6 (75%)	<0.001
**Fresh (<14 days)**	688 (76%)	36 (46%)	0.029
**Frozen (>14 days)**	66 (36%)	9 (22%)	0.029

**Table 4:** Regression analysis of risk factors.

P*	Univariate Regression Coefficient		Multivariate Regression Coefficient	P*
0.951	1.75(95%CI-54.5–57.9)	**Sex**	-	-
<0.001	-10.88(95%CI-13.15to -8.6)	**Age (years)**	-7.2(95%CI-9.5to -4.9)	<0.001
0.243	-0.003(95%CI-0.009 to 0.002)	**DTP**	-	-
<0.001	-0.56(95%CI-0.77to -0.35)	**DTS**	-0.45(95%CI-0.65to -0.25)	<0.001
<0.001	-478.8(95%CI-576.9to -380.7)	**Previous Cataract Surgery**	-392.1(95%CI-508.2to -276.1)	<0.001

Stromal clear area was defined as the central corneal diameter that was free of corneal degeneration (i.e., senile arcus, shagreen), opacities or surgical scars. It is a parameter that surgeons often use when considering corneas for optical penetrating or anterior lamellar grafts, especially for younger recipients where post-graft cosmesis is important. Based on our experience, an 8.0 mm central clear zone was the minimum diameter that surgeons were comfortable with for majority of their patients. 17% of pseudophakic corneas and 46% of phakic corneas in our series had stromal clear areas ≥ 8.0 mm (p < 0.001) Table 1. These values translated directly into the eventual grading assigned to the corneas.

20% of pseudophakic corneas were graded as A upon release compared to 68% for phakic corneas (p < 0.001). Conversely, 80% of pseudophakic corneas were graded B upon release or subsequently downgraded compared to 32% of phakic corneas (p < 0.001) Table 1. Pseudophakic corneas are downgraded more often (18% vs 10%) than phakic corneas but this was not statistically significant (p >0.001).

### Utilization rates of phakic and pseudophakic corneas

Overall, 58% of pseudophakic corneas that were deemed suitable for transplant were eventually used for surgery compared to 83% for the phakic group Table 1. Comparing the type of surgical indication, 47% of pseudophakic corneas were used for therapeutic and tectonic indications, of which anterior lamellar keratoplasty (ALK) was the predominant procedure (64%). In contrast, 84% of phakic corneas were used for optical grafts with endothelial keratoplasty (EK) being the most common procedure (41%) Table 1. ECD and clear area were two of the major determining factors in the utilization of corneas. When both criteria meet the minimum standard for optical transplant (i.e., ECD >2250 cells/mm^2^ and clear areas >8.0 mm), 75% of pseudophakic corneas were used compared to 90% of phakic group. However, when only either cell count or clear area meet the criteria, only 55% of pseudophakic corneas were utilized compared to 80% in phakic group (p <0.001). Descemet Stripping Automated Endothelial Keratoplasty (DSAEK) was the most commonly performed corneal graft in our study. The utilization rate of pseudophakic corneas meeting EK standards was 62% compared to 88% in phakic corneas (p<0.001) Table 3.

As the pseudophakic group had higher percentages of corneas with lower endothelial cell counts and smaller clear areas, 53% of these corneas were eventually frozen after 14 days of being unmatched, in comparison to the 20% freeze rate for phakic corneas (p <0.001) Table 1. Of these, 22% of pseudophakic and 36% of phakic corneas were eventually used for surgery within the 2-year grace period.

## Discussion

Our study has demonstrated statistically significant differences between phakic and pseudophakic donor corneas with regard to endothelial cell density and central clear area, two important parameters which determine the overall grade of the tissue, the type of graft the cornea is offered for, and ultimately, the utilization rate of the corneas. Pseudophakic corneas tended to be used mostly in lamellar procedures with therapeutic or tectonic indications. When donor age was analyzed as a continuous variable, the endothelial cell counts of pseudophakic donors were significantly lower than their phakic counterparts.

**Fig 1:**
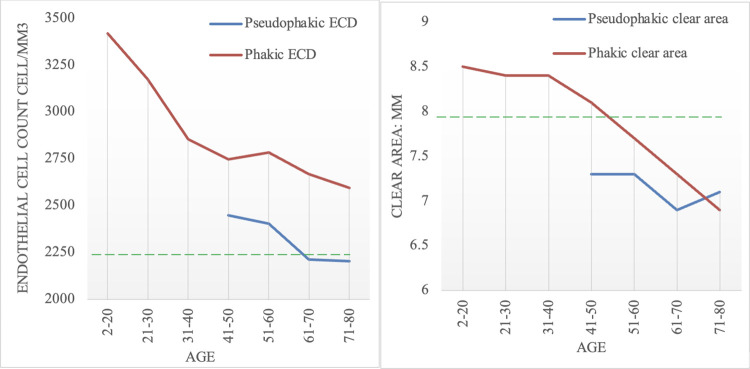
Association between age, clear area and endothelial cell density.

In a rapidly aging population like Singapore, it is not surprising that the highest proportion of cornea donors fall within the 60–70 years old range Table 5 and Fig 1. Similar to several other studies, we have demonstrated a strong correlation between age and ECD (-10.88, 95%CI-13.15to -8.6) [16–20]. Interestingly, we observed that the average age of pseudophakic donors in our study (65 ± 8 years) was relatively younger compared to those in the literature from Germany and India [4, 7]. Countries with high purchasing power parity such as Singapore have relatively easier access to healthcare resources. In addition, the overall educational status of the general population is higher and as a result, these individuals have increased expectations with regard to their visual acuity and are more likely to avail of eye care services such as cataract surgery, if required [10, 11].

**Table 5:** Association between age, clear area and endothelial cell density.

Age	Phakic	Average ECD	Clear area	Pseudophakic	Average ECD	Clear area
**2–20**	7 (1%)	3420 ± 426	8.5 ± 0.65	0	0	0
**21–30**	22 (2%)	3176 ± 427	8.4 ± 0.79	0	0	0
**31–40**	62 (7%)	2853 ± 418	8.4 ± 0.74	0	0	0
**41–50**	129 (14%)	2746 ± 417	8.1 ± 0.53	4 (4%)	2448 ± 454	7.3 ± 0.38
**51–60**	296 (33%)	2785 ± 416	7.7 ± 0.69	16 (21%)	2404 ± 516	7.3 ± 0.56
**61–70**	295 (32%)	2668 ± 416	7.3 ± 0.70	38 (49%)	2212 ± 503	6.9 ± 0.81
**71–80**	97 (11%)	2595 ± 417	6.9 ± 0.75	20 (26%)	2204 ± 506	7.1 ± 0.66

**Fig 2:**
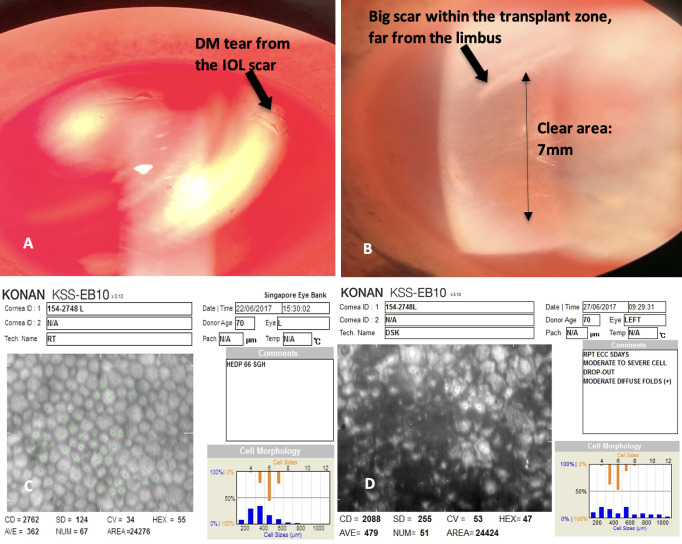
Pseudophakic cornea. **CD** (A) DM tear extended from the intra-ocular. (B) Lens scar large intra-ocular lens scar with transplantable clear zone at 7mm. (C) A pseudophakic cornea with good endothelial cell count on day 1. (D) Endothelial cell count on day 4 with diffuse area of drop out.

The Singapore Cornea Grading System was created to efficiently allocate different grades of corneas for specific types of corneal transplants. Corneas were graded A or B based on a predetermined set of parameters such as tissue procurement time, stromal clarity and endothelial cell density. The grading system allowed ease of communication between surgeons and eye banks when a cornea request was made. 68% of phakic corneas in our study received an initial tissue grading of A and 32% were considered B grade (including downgraded tissues) Table 1. The inverse was seen for pseudophakic corneas where 20% were initially graded as A and 80% were graded as B (including downgraded tissues). Under certain circumstances, a cornea initially graded as A may be downgraded to B, examples include a cornea that has not been matched by Day 8 or in instances where there is significant deterioration in endothelial cell quality during repeat specular examination Fig 2. 53% of pseudophakic and 20% of phakic corneas (p < 0.001) that go beyond the stipulated 14-day expiry date in our eye bank are placed inside a -80 Celsius freezer where they are kept for another 2 years and used mainly for therapeutic or tectonic procedures.

It is important to point out that any advantage conferred by younger age is diminished or altogether negated by the effect of cataract surgery. Our study has demonstrated the negative impact of cataract surgery on the donor endothelial cell density. Gupta *et al* and Kwon *et al* have previously demonstrated the same correlation between lens surgery and the quality of endothelial cells [5, 6]. Several studies have also shown that the post-op endothelial cell counts of graft recipients receiving pseudophakic corneas were significantly lower compared to those using phakic corneas [4, 21]. Yamazoe, K. *et al* and Hwang, H.B., *et al* observed higher rates of endothelial cell loss post-cataract surgery in patients with shallow anterior chambers [22, 23]. Epidemiologic studies conducted by Aung *et al* and by Wong *et al* have shown that shallow anterior chambers are commonly found in patients of Chinese descent, which is the largest ethnic group in Singapore. This dovetails with the high incidence of myopia observed within this group, where longer axial lengths and shallower anterior chambers are not uncommon [24, 25].

Our data also shows that less than 50% of donor corneas (46% of phakic corneas and 17% of pseudophakic corneas) in our series had stromal clear areas >8.0 mm. The most common reason for the small clear areas can be attributed to arcus senilis. However, even with age adjusted statistical analysis, pseudophakic corneas generally have smaller clear areas Table 5. This can be attributed to the smaller cornea diameter in Asian population in general and relatively paracentral location of the clear cornea incisions created during cataract surgery could aggravate the originally smaller eyes [12–14, 26–28]. Aside from effectively limiting the centraI clear zone, the clear cornea incisions also induce morphological changes to the corneal stroma and endothelium which ultimately influence the final grading and utilization of corneas.

Over the past two decades, corneal transplant surgeries have shifted from full thickness procedures to selective lamellar replacement surgery. This revolutionary change is especially evident in EK which has supplanted PK as the preferred procedure for conditions like pseudophakic bullous keratopathy and Fuch’s endothelial dystrophy [29–32]. The Singapore Eye Bank registry shows that over 50% of grafts performed in 2019 were EK. The minimum standards for an EK quality cornea in our eye bank include a maximum graft to surgery interval of 7 days, an ECD of at least 2500 cells/mm^2^ with little to no endothelial cell drop, and the absence of any pathology in the Descemet’s membrane within the planned graft zone. Only 37% of pseudophakic corneas in our series had ECD >2,500 cells/mm^2^ compared to 77% for the phakic group. Utilization rates for this subset were 62% for pseudophakic corneas and 88% for phakic corneas. In the subset of pseudophakic corneas with ECD>2,500 cells/mm^2^ but were not utilized, the most common reason for non-usage was the presence of clear cornea incisions (30%). In our series, the incisions were encroaching upon the paracentral cornea, effectively limiting the central corneal area to approximately 6.0 to 6.5 mm. Some surgeons regard the incision as a potential weak spot in the cornea which may extend into a Descemet’s tear or detachment and are unwilling to use the tissue. This could pose a significant problem given the current shortage of donor tissue. A potential solution to maximize use of these corneas would be to perform asymmetric trephination of donor tissue, specifically for use in descemet membrane endothelial keratoplasty (DMEK) [33, 34].

Our data suggests that pseudophakic corneas in general have a lower probability of meeting the parameters required for use in optical transplants, and with stringent requirement from surgeons this poses a real challenge to the eye bank when the greatest demand from our surgeons are corneas that can be used for EK. Unmatched and unused tissue will severely impact the eye bank’s long-term financial sustainability. Our study shows that 53% of all pseudophakic corneas and 20% of phakic corneas retrieved were frozen for emergency tectonic or therapeutic uses. At the end of the 2-year grace period, only 22% of pseudophakic and 36% of phakic corneas were eventually used for surgery.

From our own experience, a surgeon will gravitate towards a cornea perceived as being “better” and under certain circumstances, surgeon bias against pseudophakic corneas does exist. Such “cherry picking” practices can be averted by maintaining open channels of communication between the eye bank and the surgeons. It should be made clear that any cornea that passes the rigid quality controls imposed by the eye bank and deemed suitable for a particular type of corneal procedure should by right, be accepted regardless of the donor’s lens status. Despite, advancement in cataract surgery techniques and instrumentation will hopefully further minimize corneal damage and increase the utilization rate of pseudophakic corneas [13].

Surgeon bias aside, this does not detract from the fact that a true disparity exists between phakic and pseudophakic cornea utilization rates and that this in turn is a consequence of the measurably lower cell counts and clear areas seen in pseudophakic corneas. Continuing our practice of accepting pseudophakic donors needs to be revisited and if needed, recalibrated. Otherwise, we will continue to have a high non-utilization rate which cumulatively will affect the eye bank’s self-sustainability efforts. It would be unrealistic to reject all pseudophakic donors across the board, bearing in mind that we expect an increase in the number of pseudophakic corneas in our local donor pool in the coming years. Additionally, some studies have supported raising the maximum donor age limit in order to expand the donor pool and fill the ever-increasing demand for corneas [35–38]. A more measured response would perhaps be to exclude bilateral pseudophakic donors beyond a certain age group. We propose excluding pseudophakic donors 70 years old and above as they have been shown to have average endothelial cell densities below 2250 cells/mm^2^ and transplantable stromal clear area below 8mm. Such a proposal may help the eye bank strike a balance between sustaining donor numbers and optimizing utilization rates, when alternative allocation of tissues for other purposes such as training, and research is not fully established [39].

Our study reflects the difficulties small scale eye banks face in trying to obtain sufficient numbers of corneas to meet demand while ensuring efficient and optimal utilization of tissue. The main limitation of our study is the significant disproportion in the sample populations for the two study groups, and this insignificant sample size could have a potential risk of type II error. This however is a reflection of the prevalence rates in the general population as our eye bank currently does not actively exclude donors merely on the basis of previous cataract surgery. As this study was performed at a single eye bank in Singapore, the trends observed in this study may not reflect the general trend in other geographical locations where corneal transplants are performed. It will require a larger scale study in multi-centred / multi-national approach.

## Conclusion

Pseudophakic corneas generally have poorer cell quality compared to phakic corneas, leading to an overall lower utilization rate for optical grafts. A timely reassessment of the eye bank’s internal acceptance policy with regard to donor lens status exclusion criteria is required in order to address this issue and to ensure that utilization of all recovered corneas are optimized. Clear communication with surgeons is also important to ensure that corneas that fulfil the quality criteria for surgery should be accepted, regardless of donor lens status.

## Supporting information

S1 Data (XLSX)
